# Evidence for superior encoding of detailed visual memories in deaf signers

**DOI:** 10.1038/s41598-022-13000-y

**Published:** 2022-05-31

**Authors:** Michael Craig, Michaela Dewar, Graham Turner, Trudi Collier, Narinder Kapur

**Affiliations:** 1 Department of Psychology, Faculty of Health and Life Sciences, Northumbria University, Newcastle upon Tyne, UK; 2 Memory Lab, Department of Psychology, School of Social Sciences, Heriot-Watt University, Edinburgh, UK; 3 Centre for Translation and Interpreting Studies in Scotland, School of Social Sciences, Heriot-Watt University, Edinburgh, UK; 4 Division of Psychology and Language Sciences, Department of Clinical, Education and Health Psychology, Faculty of Brain Sciences, University College London, London, UK

**Keywords:** Psychology, Human behaviour

## Abstract

Recent evidence shows that deaf signers outperform hearing non-signers in some tests of visual attention and discrimination. Furthermore, they can retain visual information better over short periods, i.e., seconds. However, it is unknown if deaf signers’ retention of detailed visual information is superior following more extended periods. We report a study investigating this possibility. Our data revealed that deaf individuals outperformed hearing people in a visual long-term memory test that probed the fine detail of new memories. Deaf individuals also performed better in a scene-discrimination test, which correlated positively with performance on the long-term memory test. Our findings provide evidence that deaf signers can demonstrate superior visual long-term memory, possibly because of enhanced visual attention during encoding. The relative contributions of factors including sign language fluency, protracted practice, and neural plasticity are still to be established. Our findings add to evidence showing that deaf signers are at an advantage in some respects, including the retention of detailed visual memories over the longer term.

## Introduction

Recent research shows that deaf sign language users can outperform hearing non-signers in some tests of visual cognition. These ‘deaf gains’^1^ have been reported chiefly in tests of visual attention^2,3^, and especially the detection of details in the periphery of the visual field and motion cues^3–5^. Deaf signers also demonstrate a superior ability to discriminate between subtly different visual stimuli, including photos of everyday objects, real-world scenes, and faces^6–8^. Furthermore, they show a heightened ability to identify subtle discrepancies in facial emotion^8,9^.

These gains in the visual modality are not restricted to attention and discrimination. Evidence shows that the retention of visuospatial codes in short-term (working) memory over the shorter term, i.e., seconds, is superior in deaf signers^10–12^. Enhanced attention for presented visual stimuli is proposed to account for these gains in short-term memory^4,10^. It is however challenging to ascertain the relative contributions of, and interactions between, deafness and sign language fluency, as well as the possible contributions of other factors including education, family history, protracted practice, and neural reorganisation^4^.

Nevertheless, irrespective of the underpinnings of superior visual cognition in deaf signers, it is not yet known if their retention of visual information is superior following more extended periods, i.e., minutes to hours. If so, this would indicate that deaf gains in visual cognition extend to the declarative long-term memory system^13,14^. Evidence for enhanced visual *long-term* memory in deaf signers would not only be of scientific and societal interest; it could also reveal niches for deaf people in the workplace, akin to the recruitment of so-called ‘super recognisers’ to the police force. To this end, we examined whether deaf signers’ visual long-term memory is superior to that of hearing non-signers.

We applied a variant of the robust and sensitive ‘Mnemonic Similarity Task’ (MST)^15^: deaf signers (n = 20) and hearing non-signers (n = 20) viewed photos of 50 everyday items, and, following a filled 10-min delay (scene discrimination task), completed a memory test that probed their ability to remember fine details of the photos. In this test, participants were presented a total of 75 photos, which comprised (i) 25 photos that were identical to those viewed during encoding (targets), (ii) 25 photos that were visually similar to those viewed during encoding (lures), and (iii) 25 photos that were brand new (foils). For each photo, participants were asked whether it was *old*, *similar*, or *new*. Using the responses in the MST memory test, we calculated a standard recognition score—to measure gist memory—and a Lure Discrimination Index (LDI) score—to measure the fine detail of memories. It was hypothesised that, should deaf gains extend to the retention of visual memories over the longer term, deaf participants should demonstrate superior long-term memory than hearing participants in our variant of the MST.

Participants also completed a short-term memory test, face-discrimination test, and sensory imagery questionnaire to explore potential associations between previously reported group differences in these visual domains and possible group differences in our long-term memory test. Due to ceiling effects, we do not consider the visual short-term memory and face-discrimination test further (see Supplementary Information for a full report of these tests and results). Finally, participants completed an autobiographical memory questionnaire to explore possible differences in subjective reports of everyday memory abilities. All study materials, tasks and data are available in the Open Science Framework repository, https://osf.io/rhs4p/.

## Results

Data from two participants (deaf: N = 1; hearing: N = 1) were removed as outliers because they performed more than two standard deviations from their respective group means on the long-term memory test. Thus, the following analyses report data from N = 38 participants (deaf: N = 19; hearing: N = 19).

### Background measures

Our deaf and hearing groups were matched in their age (deaf: M = 43.11 years, SD = 9.08 years, range = 28–57 years; hearing: M = 37.58 years, SD = 11.47 years, range: 19–56 years; F(1,36) = 2.71, *p* = 0.108, ηρ^2^ = 0.070), gender ratio (deaf: 9F:10M; hearing group: 10F:9M; Fisher exact test *p* = 1.000), and years spent in education (deaf: M = 17.74 years, SD = 4.48 years, range: 10–27 years; hearing group: M = 15.74 years, SD = 1.79 years, range: 13–19 years; F(1,36) = 3.26, *p* = 0.079, ηρ^2^ = 0.083).

### Long-term memory test

*Encoding* Performance in the encoding phase was matched between groups. We found no differences in the percentage of trials that participants responded to (deaf: M = 93.40%, SD = 0.05%; hearing: M = 93.60%, SD = 0.07%; (F(1,36) = 0.01, *p* = 0.915, ηρ^2^ = 0.000). Further to this, comparable response times to encoding trials were observed (deaf: M = 1.09 s, SD = 0.14 s; hearing: M = 1.05 s, SD = 0.20 s; F(1,36) = 0.64, *p* = 0.428, ηρ^2^ = 0.018).

**Figure 1:**
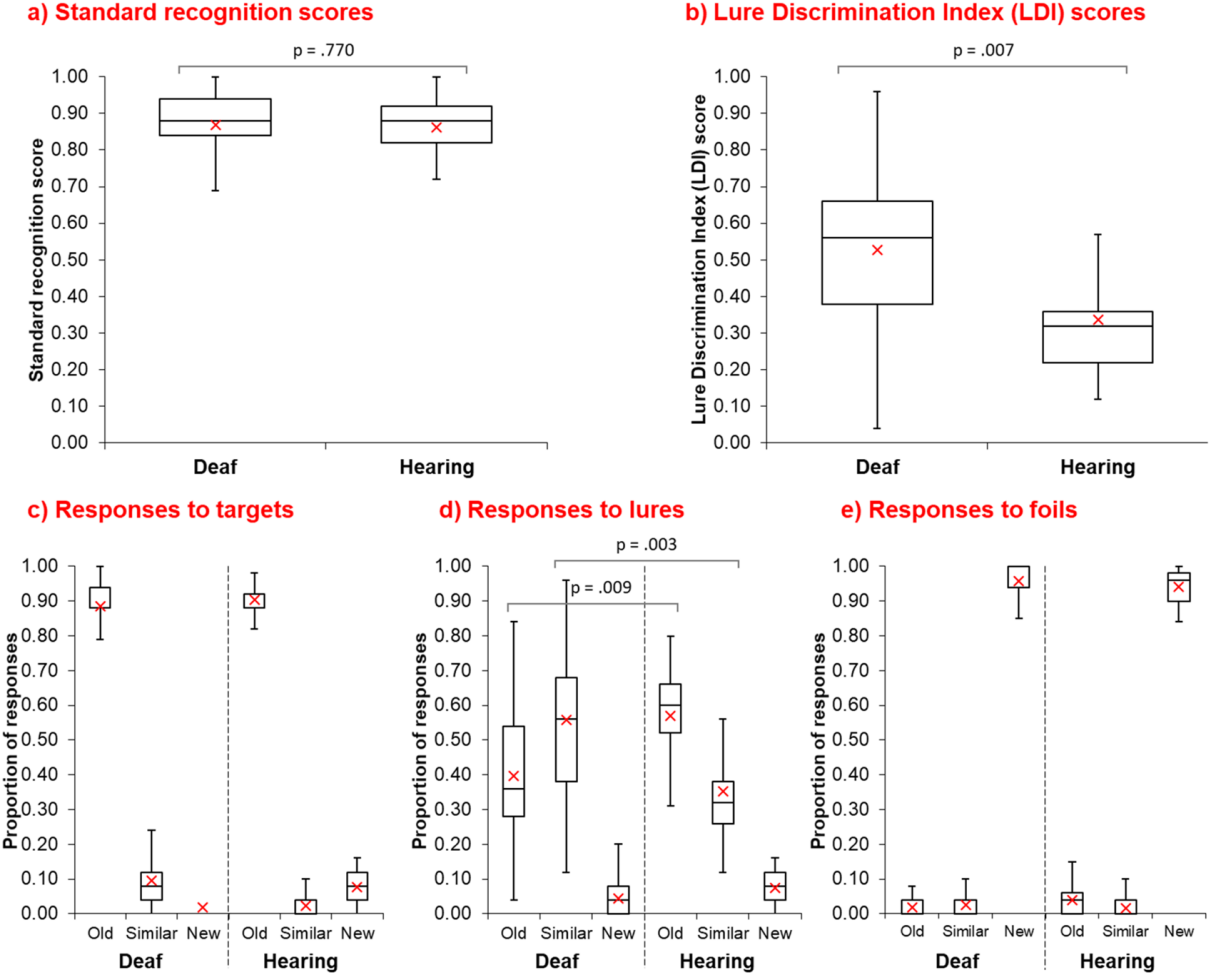
Long-term memory test performance. Box-whisker plots showing (**a**) standard recognition scores for the deaf group (N = 19) and hearing group (N = 19), (**b**) Lure Discrimination Index (LDI) scores for the deaf group and hearing group, and (**c**–**e**) the proportion of ‘old’, ‘similar’, and ‘new’ responses to targets (**c**), lures (**d**), and foils (**e**) for the deaf and hearing groups. The solid centre line in each box shows the median score for that group. The upper and lower boundaries of boxes represent the interquartile ranges. Box whiskers show the upper and lower quartiles of scores. The red asterisk in each box shows the mean.

*Testing* Fig. 1 shows data from the testing phase. Standard recognition scores (Fig. 1a) were comparable between groups (deaf: M = 0.87, SD = 0.10; hearing group: M = 0.86 SD = 0.07; F(1,36) = 0.09, *p* = 0.770, ηρ^2^ = 0.002). We did however find a significant group difference in LDI scores (Fig. 1b), which provided a measure of participants’ ability to retain detailed representations of encoded photos (deaf: M = 0.53, SD = 0.24; hearing group: M = 0.34, SD = 0.170; F(1,36) = 8.26, *p* = 0.007, ηρ^2^ = 0.187). This superior LDI performance in the deaf group remained significant when including age as a covariate (F(1,35) = 8.67, *p* = 0.006, ηρ^2^ = 0.199).

Figure 1c-e provides a breakdown of participants’ responses in the testing phase, where the proportion of *‘old’*, *‘similar’* and *‘new’* responses to targets (Fig. 1c), lures (Fig. 1d), and foils (Fig. 1e) are shown. In keeping with recent work^16^, to probe further the significant difference in LDI scores, we conducted a repeated measures ANOVA using the proportion of ‘old’ and ‘similar’ responses to lures, as this is where any differences in participants’ ability to detect subtle differences in the visual appearance between targets and lures should appear. The repeated measures ANOVA revealed no significant main effect of response type (‘old’ vs. ‘similar’) (F(1,36) = 0.30, *p* = 0.590, ηρ^2^ = 0.008), but we did find a significant main effect of group (hearing vs. deaf) (F(1,36) = 5.09, *p* = 0.030, ηρ^2^ = 0.124) and interaction between group and response type (F(1,36) = 8.99, *p* = 0.005, ηρ^2^ = 0.200). Pairwise comparisons revealed that this interaction was due to significant group differences in the proportion of incorrect ‘old’ responses to lures (t(36) =  − 2.77, *p* = 0.009) and correct ‘similar’ responses to lures (t(36) = 3.17, *p* = 0.003), where deaf participants made fewer incorrect ‘old’ responses to lures and more correct ‘similar’ responses to lures, relative to the hearing group (see Fig. 1d).

Response times during the testing phase differed significantly between groups (F(1,36) = 9.20, *p* = 0.004, ηρ^2^ = 0.204). Deaf individuals (M = 2.69 s, SD = 0.72 s) were slower to respond than their hearing counterparts (M = 1.93 s, SD = 0.81 s). When breaking items down into targets, lures, and foils, we found a significant main effect of item type (F(2,72) = 10.51, *p* < 0.001, ηρ^2^ = 0.226). This was because, overall, participants were slower to respond to lures (M = 2.57 s, SD = 1.11 s) than both targets (M = 2.26 s, SD = 0.84 s, t(36) =  − 3.72, *p* = 0.002) and foils (M = 2.10 s, SD = 0.79 s, t(36) = 3.45, *p* = 0.004). Both significant findings remained following Bonferroni corrections for multiple comparisons (alpha level of 0.050/three comparisons = corrected alpha level of 0.017). There was no significant interaction between group (deaf vs. hearing) and item type (targets vs. lures vs. foils) indicating that this trend was similar in both groups (F(2,72) = 2.39, *p* = 0.099, ηρ^2^ = 0.062).

Pearson correlations revealed no significant relationship between response times and standard recognition or LDI scores (all *p* > 0.207).

### Scene discrimination task

Comparison of raw discrimination scores revealed that deaf individuals identified significantly more subtle differences in ‘different’ trials (deaf: M = 0.81, SD = 0.12; hearing: M = 0.71, SD = 0.11; F(1,36) = 6.74, *p* = 0.014, ηρ^2^ = 0.158). This finding did not change when using corrected discrimination scores (see Methods) that account for response bias (deaf: M = 0.77, SD = 0.13; hearing: M = 0.67, SD = 0.12; F(1,36) = 5.16, *p* = 0.029, ηρ^2^ = 0.125).

Pearson correlation analyses revealed a significant correlation between the long-term memory test LDI score and the scene discrimination task raw score (r = 0.34, *p* = 0.037) and the scene discrimination task corrected score (r = 0.33, *p* = 0.042).

### Questionnaires on imagery and autobiographical memory

Table 1 shows total scores from the Vividness of Visual Imagery Questionnaire (VVIQ)^17^, Survey of Autobiographical Memory (SAM) questionnaire^18^, and Plymouth Sensory Imagery Questionnaire (PSIQ)^19^ for the deaf and hearing groups. Significance values from independent t-tests are also reported in the table. We found no significant group differences in total scores for the VVIQ (t(36) =  − 0.93, *p* = 0.360), SAM (t(36) =  − 0.36, *p* = 0.723), or PSIQ (t(36) =  − 0.63, *p* = 0.534). When breaking SAM total scores down into sub-scores, we found no significant group differences in Episodic, Semantic, Spatial, or Future thinking scores (all *p* > 0.165). We also found no significant between-group differences in most PSIQ sub-scores (Imagination, Smell, Taste, Touch, Bodily sensation, Feeling; all *p* > 0.142). We did however find a significant group difference in the Sound sub-score (t(36) =  − 5.36, *p* < 0.001), which remained following Bonferroni-correction for multiple comparisons (alpha level of 0.05/7 comparisons = corrected alpha level of 0.007). This significant difference was due to participants in the hearing group reporting being better able to internally imagine a range of everyday sounds (e.g., a cat’s meow, children playing).

**Table 1:** Questionnaire data for participants in the deaf and hearing groups.

Questionnaires and sub-sections	Group		Significance value
	Deaf	Hearing	
VVIQ Total (/80)	60.84 (9.48)	63.53 (8.30)	*p* = .360
**SAM Total (/130)**	85.10 (13.91)	86.53 (10.37)	*p* = .723
SAM Episodic (/40)	23.63 (5.35)	25.68 (10.37)	*p* = .197
SAM Semantic (/30)	18.47 (4.93)	19.10 (3.98)	*p* = .667
SAM Spatial (/30)	22.68 (4.84)	20.84 (2.97)	*p* = .166
SAM Future (/30)	20.32 (4.50)	20.89 (3.18)	*p* = .650
**PSIQ Total (/350)**	258.37 (47.29)	266.74 (33.80)	*p* = .534
PSIQ Appearance (/50)	42.42 (6.45)	40.47 (6.62)	*p* = .365
PSIQ Sound (/50)	17.10 (17.72)	40.37 (6.60)	*p* < .001*
PSIQ Smell (/50)	40.58 (9.75)	37.84 (5.94)	*p* = .303
PSIQ Taste (/50)	39.47 (11.47)	34.89 (6.79)	*p* = .143
PSIQ Touch (/50)	39.16 (11.60)	38.42 (6.34)	*p* = .809
PSIQ Bodily sensation (/50)	39.89 (8.05)	37.37 (3.93)	*p* = .227
PSIQ Feeling (/50)	39.74 (8.35)	37.37 (5.74)	*p* = .314

Data are shown from the Vividness of Visual Imagery Questionnaire (VVIQ) (Marks^17^), Survey of Autobiographical Memory (SAM) (Palombo et al.^18^), and Plymouth Sensory Imagery Questionnaire (PSIQ) (Andrade et al.^19^). Total scores and sub-scores for the SAM and PSIQ are shown. Data in parentheses show standard deviations. Independent *t*-test comparisons were used to probe possible between-group differences. Bold text shows *p* values that reached the threshold for significance. An accompanying asterisk (*) indicates that a significant *p* value remained following Bonferroni correction for multiple comparisons.

Pearson correlation analyses revealed no relationships between total scores or sub-scores from our questionnaires and performance in the long-term memory test (recognition and LDI) (all *p* > 0.112).

## Discussion

Our data reveal that deaf signers outperformed hearing non-signers in a visual long-term memory test that required the discrimination between recently encoded photos and similar lures. Deaf participants also demonstrated superior performance in a scene discrimination task, which correlated significantly with that on the visual long-term memory test. Scores in autobiographical memory and internal sensory imagery questionnaires were comparable and did not correlate with the visual long-term memory scores.

The findings of our study add to a growing body of evidence demonstrating enhanced visual cognition in deaf signers^4^, though some findings are mixed^12^. Pertinent to our study, we reveal that deaf gains^1^ in visual cognition are not restricted to attention, discrimination, and the retention of visual codes over the shorter term, i.e., seconds. Our data show that deaf signers can also demonstrate a superior ability to retain detailed visual memories over the longer term, i.e., minutes.

Why did our deaf group outperform the hearing group in our long-term memory test? Our study was designed as a first-step investigation to explore *whether* deaf signers have superior visual long-term memory skills, so we cannot provide a definitive answer as to *why* this effect occurred. Nevertheless, our methods and the collected data offer some insights.

Our deaf and hearing groups were matched in age, gender, and years in education. These factors are therefore unlikely to account substantially for superior memory scores in deaf participants. Moreover, the two groups demonstrated similar abilities in their internal sensory imagery and everyday memory abilities, and scores in these measures did not correlate with memory. Thus, group differences in visual imagery and everyday memory abilities are unlikely to account for our findings.

The LDI measure of our long-term memory test probed participants’ ability to discriminate between encoded targets and similar lures *after a 10-min filled retention interval*. Therefore, this test does not merely depend on the online discrimination of similar visual codes. Instead, performance in the LDI measure depends on the *quality* of visual representations stored in long-term memory. Higher-quality visual representations should allow for superior discrimination at a delayed stage, thus higher LDI scores^16^. Superior LDI scores in deaf participants, therefore, indicate that they retained higher quality visual long-term memories.

Can we identify the long-term memory process(es) that were enhanced in deaf participants and resulted in higher quality memories? In keeping with existing work^16^, the design of our test meant that memory for encoded targets was not probed at an immediate or intermediate stage. Therefore, it is challenging to establish precisely which, if any, memory processes were enhanced. One possibility is that deaf participants retrieved encoded memories better. However, this is unlikely as we observed (i) no speed-accuracy trade-offs during testing, and (ii) all participants performed near ceiling in the standard recognition measure of the long-term memory test, where performance was comparable between groups. These findings demonstrate that all participants stored (at least) gist representations for most encoded memories and were able to retrieve these memories successfully.

A further possibility is that the early consolidation (i.e., strengthening) of the new memory traces during the 10-min delay was superior in deaf participants, thus resulting in higher-quality memories. Unfortunately, the design of our study does not allow us to test this, nor does it power us to eliminate group differences in consolidation interference, for example, from sensory input associated with the scene discrimination task that was completed during the 10-min retention interval^16^.

In keeping with recent findings, we hypothesise that the superior memory quality in deaf participants resulted from enhanced encoding capabilities. Although our deaf and hearing groups’ performance on the judgement making task was matched, it is plausible that the two groups encoded the photos differently. Research demonstrates enhanced visual attention in deaf signers^2,3^, and this is proposed to underly deaf gains in visual cognition tasks^4^. This proposal is supported by eye-tracking research demonstrating increased exploration of visual stimuli in deaf signers^20^. In hearing people, eye-tracking research shows that the number of fixations during encoding positively predicts subsequent performance in lure discrimination tests, like the one used in our study^21^. It is thought that a larger number of fixations to a stimulus during encoding increases the likelihood of a higher-quality memory trace being formed^22^. Thus, we propose that enhanced attention in our deaf participants, possibly through a higher number of fixations, led to the formation of memory traces that were rich in detail. These detail-rich memory traces should have been more discriminable from similar lures, thus resulting in superior performance in our LDI measure. Future work could test this proposal using eye-tracking methods.

We also acknowledge that we cannot rule out a possible contribution of superior working memory abilities, for example, in the mental rotation of previously encoded stimuli for comparison against items presented in the testing phase. Evidence indicates sign language users can demonstrate superior mental rotation abilities for visual stimuli^23^. In our study, some lure items were rotated versions of their encoded counterparts though lures also differed in their size, colour, and content. For this reason, superior mental rotation is unlikely to fully explain superior LDI scores, but a partial contribution is possible. Controlling for such factors in future work would be beneficial. Deaf participants also significantly outperformed hearing participants in the identification of subtle differences in the scene discrimination task. Superior performance in this task, in the LDI measure of the long-term memory test, and the positive correlation between these two measures, could suggest a general superiority in deaf participants in discriminating between similar visual codes. This proposal resonates with previous findings demonstrating superior visual discrimination in deaf signers^6,8,24^. It is possible that these findings can also be explained by enhanced visual attention and a greater number of fixations in deaf individuals. Deaf gains in visual attention appear to be most prominent for detecting subtle visual details, for example, minor motion cues or information in the periphery^4^. Thus, enhanced visual attention in the deaf participants may account for superior performance in both the scene discrimination task and LDI measure of the long-term memory test. Alternatively, or in addition, it is possible that the combined visual load of the scene discrimination task and long-term memory test was of greater cognitive burden for hearing than deaf participants and was detrimental to their performance in both tasks. Superior visual attention in deaf individuals may have reduced the burden of this visual load.

We found that deaf participants were slower to respond to testing phase trials in our long-term memory test. There was no relationship between response times and memory performance in either group. One possible explanation for extended response times is that deaf participants required longer to process visual information. This resonates with previous findings using alpha numeric stimuli, where deaf participants were approximately 100 ms slower to respond than hearing participants because these stimuli are processed slower in deaf individuals^3^, though outcomes are mixed, and some studies show faster visual processing and responses in this population^2^. It has been suggested that these contrasting findings may be because visual cognition is not uniformly enhanced in deaf individuals^4^, and such variations may be recorded differently in various tests of visual cognition. Another possibility is that deaf participants may have been more motivated to provide correct responses, which resulted in more extended periods of deliberation and response times. If true, the lack of speed-accuracy trade-offs in the long-term memory test suggests that this was of no benefit to performance. These possibilities should be explored in future work.

Some significant limitations should be considered with our findings. First, given the relatively small number of deaf signers in the population—N =  ~ 1000 in the local Edinburgh area of Scotland, UK^25^ and the challenges associated with recruiting “hard-to-reach” populations, our sample and study power were modest. A posthoc power analysis for the crucial difference in LDI scores provided a power value of 1 − β = 0.624. Further replications in larger samples are therefore warranted. Second, to encourage the recruitment of deaf signers, we applied relatively broad inclusion criteria. This resulted in a relatively wide range of ages in both groups. The adverse effects of ageing on memory, including the LDI applied here, are well known^15,26^. However, superior memory in the deaf participants remained significant after controlling for the effect of age, and in any case, our deaf participants were, on average, older than our hearing participants. Therefore, the superior memory in our deaf group cannot be explained away by age. However, future work should consider using a sample with a narrow age range to reduce age-related contributions to memory. Third, to minimise participant fatigue, we did not empirically measure hearing ability or fluency in sign language, and we did not record whether participants used a form of hearing aid. To understand the contribution of sign language fluency to our findings, it would have been advantageous to include four participant groups: deaf signers, deaf non-signers, hearing signers, and hearing non-signers. Because of these limitations, it is challenging to ascertain the relative contributions or interactions of deafness, education and family history, protracted practice, neural reorganisation, and fluency in sign language, which may have all contributed to our findings. It is possible that individual differences in these factors might explain some of the broad variability in LDI scores in the deaf participants. Nevertheless, we believe this study can stimulate comprehensive work investigating deaf gains in visual memory, and, irrespective of the underlying mechanisms, our finding of superior visual long-term memory in deaf signers is of broad interest and can have a societal impact.

What are the implications of our study? Our findings raise questions for future work investigating deaf gains and contribute to challenging the stigma that deaf people are disadvantaged and any resulting prejudice^27^. In fact, our data add to evidence showing that deaf people are at an advantage in some respects. This impact could be felt at a societal level, for example, regarding employment opportunities that utilise deaf gains. The recruitment of ‘super recognisers’ in police forces is an intriguing example of this^28,29^ though impact could be far reaching and potentially extend to health and education settings, and the arts.

## Materials and methods

### Participants

We recruited 20 deaf participants (10 females, 10 males; age: M = 43.55 years, SD = 9.06, age range: 28–57 years) and 20 hearing participants (11 females, 9 males; age: M = 37.35 years, SD = 11.21, age range: 19–56 years) from the Edinburgh area of Scotland, UK. To encourage recruitment of deaf participants, we used relatively broad inclusion criteria for this study. All participants had normal or corrected-to-normal visual acuity and no known psychiatric or neurological disorders. Seventeen deaf participants were born deaf, and three were deaf because of meningitis (N = 2, acquired at 1 and 2 years old) or measles (N = 1, acquired at age 1.5 years). All deaf participants were bilingual and fluent in written English and British Sign Language (BSL)^30,31^. Most deaf participants learned BSL as children (< 18 years old, N = 14), and the remaining participants learned BSL in adulthood (≥ 18 years old, N = 6). The mean age of onset for BSL acquisition was 10.80 years (SD = 9.71 years, median = 6.50 years, range = 2–31 years). On average, deaf participants started to learn BSL 32.75 years before completing the present study (SD = 14.30 years, median = 35.00 years, range = 10–54 years). Hearing participants were self-reportedly monolingual in written and spoken English and had little or no exposure to BSL.

### Design

We applied a between-subjects design to examine possible differences in visual long-term memory. The study was delivered in the lab within a single session lasting approximately 90 min. Ethical approval was granted from Heriot-Watt University’s School of Social Sciences Research Ethics Committee (Ref: 2017-504), and all procedures adhered to the appropriate ethical principles for human research, including the revised Helsinki Declaration. Informed consent was obtained from all participants.

### Materials

The long-term memory test and scene discrimination task were developed and run using the psychological experimental research software, PsychoPy (version 3.0.0)^32^, and presented using a standard 19-inch widescreen computer monitor. The questionnaires were in pen and paper format. Instructions for all tests and questionnaires were presented via PsychoPy using the computer monitor. BSL instruction videos were developed for all computerised tests, questionnaires, and briefing and debriefing components of the study. These videos were developed according to existing guidelines^33^, which included forward and reverse translations. Throughout the study, we presented both written English instructions and BSL instructions side-by-side on the computer screen to all participants, so they had the option of using one or both media. If the participant required further instructions, details were provided by the experimenter in BSL for deaf participants and spoken English for hearing participants.

### Procedure

#### Long-term memory test

**Figure 2:**
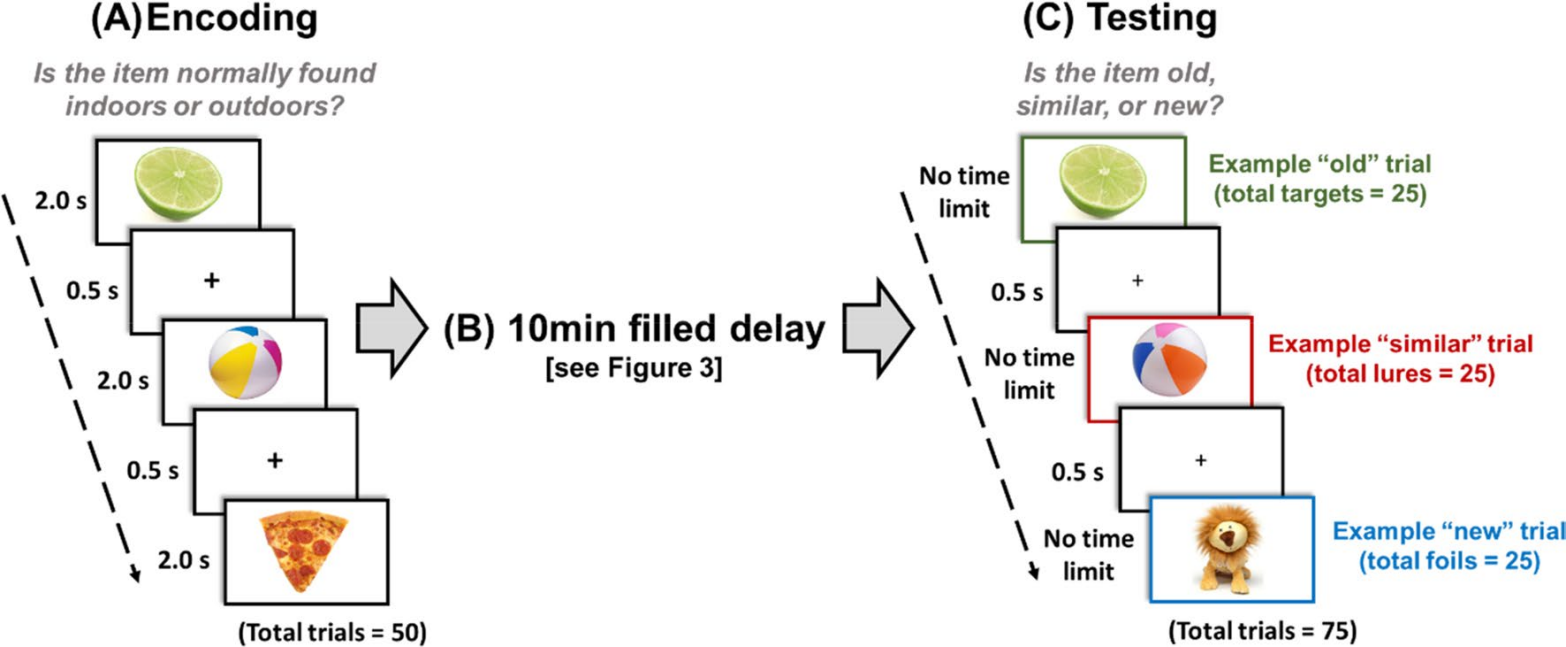
Long-term memory test procedure. Participants underwent three phases: (**A**) encoding, (**B**) 10-min filled delay, and (**C**) testing. During encoding, participants were presented 50 photos of a range of unique everyday items from the Mnemonic Similarity Task (Stark et al.^15^). Participants incidentally encoded these items via a judgment making task, where they were required to respond whether a presented item would typically be found indoors or outdoors. Each item was presented for 2 s and was followed by a 0.5-s inter-stimulus crosshair (+). Following encoding, participants experienced a filled 10-min delay condition, where they completed a scene-discrimination task (see Fig. 3). In the subsequent testing phase, participants were presented 25 of the ‘old’ items presented during encoding (targets), along with 25 ‘similar’ items that were visually similar objects from the same semantic category to the remaining 25 items presented during encoding (lures), and 25 ‘new’ items that were visually and semantically different to the items presented during encoding (foils). The figure shows examples of target, lure, and foil trials. There was no limit on the time to respond during testing.

Figure 2 provides an overview of the long-term memory test procedure. This was a modified (shortened) version of the Mnemonic Similarity Task^15,34^ combined with a delay task that was designed initially for studies on consolidation^16,35^. The procedure comprised three phases: encoding, 10-min filled delay, testing.

*Encoding* Participants were presented with a total of 50 photos of a range of unique everyday items (see Fig. 2). Each item was presented as a standalone item on a white background. As in previous work^15,16^, each item was presented for two seconds, with an inter-stimuli crosshair (+) appearing in the centre of the screen for 0.5 s (total duration of encoding phase = 150 s). Thus, all participants received identical treatment and exposure to stimuli during encoding. When presented with an item, participants were required to judge whether they believed it would typically be found indoors or outdoors. For example, if presented with a photo of a sofa, this item would typically be found indoors. However, if presented with a photo of a tree, this item would typically be found outdoors. Participants input their responses via the ‘z’ (indoors) and ‘m’ (outdoors) keys on the computer keyboard. They were instructed that some items may be ambiguous (i.e., may be found indoors and outdoors), but they should respond as quickly as possible and respond with their first instinct. We used an incidental encoding procedure to reduce the likelihood of mnemonic strategies pertaining to presented stimuli.

*10-min filled delay* Participants completed the scene discrimination task (see full description below) for 10 min. This test acted as a filler task for the 10-min retention interval of the long-term memory test^16,35^.

*Testing* In this phase, participants were presented sequentially 75 photos of everyday items. Of these photos, 25 were identical to those presented during encoding (*old* targets), 25 were subtly different to those presented during encoding (*similar* lures), and 25 were brand new items (*new* foils). See Fig. 2 for examples of old, similar, and new photos. Participants used the computer keyboard to provide an ‘old’ (visually identical target item; ‘z’ key), ‘similar’ (visually similar lure item; ‘v’ key), or ‘new’ (new foil item; ‘m’ key) response using the index finger of their dominant hand. We did this to avoid potential response biases, e.g., left hand to ‘z’ key and right hand to ‘m’ key. There was no time limit to respond; participants were asked to respond as accurately as possible, not as quickly as possible. Throughout the testing phase, written instructions regarding which keys corresponded to which response (e.g. ‘z’ = old) were always shown on the computer screen.

Target items were always the oddly numbered items presented during the encoding phase (i.e., 1, 3, 5, 7, 9,…etc.), and lure items were related to the even number items presented during the encoding phase (i.e., 2, 4, 6, 8, 10,…etc.). The 25 target and 25 lure items were then combined with 25 foil items and ordered randomly using the random number generation tool in Microsoft Excel. The same random order of items was presented to all participants.

From participants’ responses in the long-term memory test, two key measures were calculated: (i) a standard recognition score and (ii) a Lure Discrimination Index (LDI) score^15^. The standard recognition score reflects participants’ ability to endorse targets and reject foils [(proportion of old responses to targets) minus (proportion of old responses to foils)]. The LDI score reflects participants’ ability to discriminate between targets and similar lures [(proportion of similar responses to lures) minus (proportion of similar responses to foils)]. The time that it took participants to respond to trials during encoding and testing was also recorded for analyses.

#### Scene discrimination task

**Figure 3:**
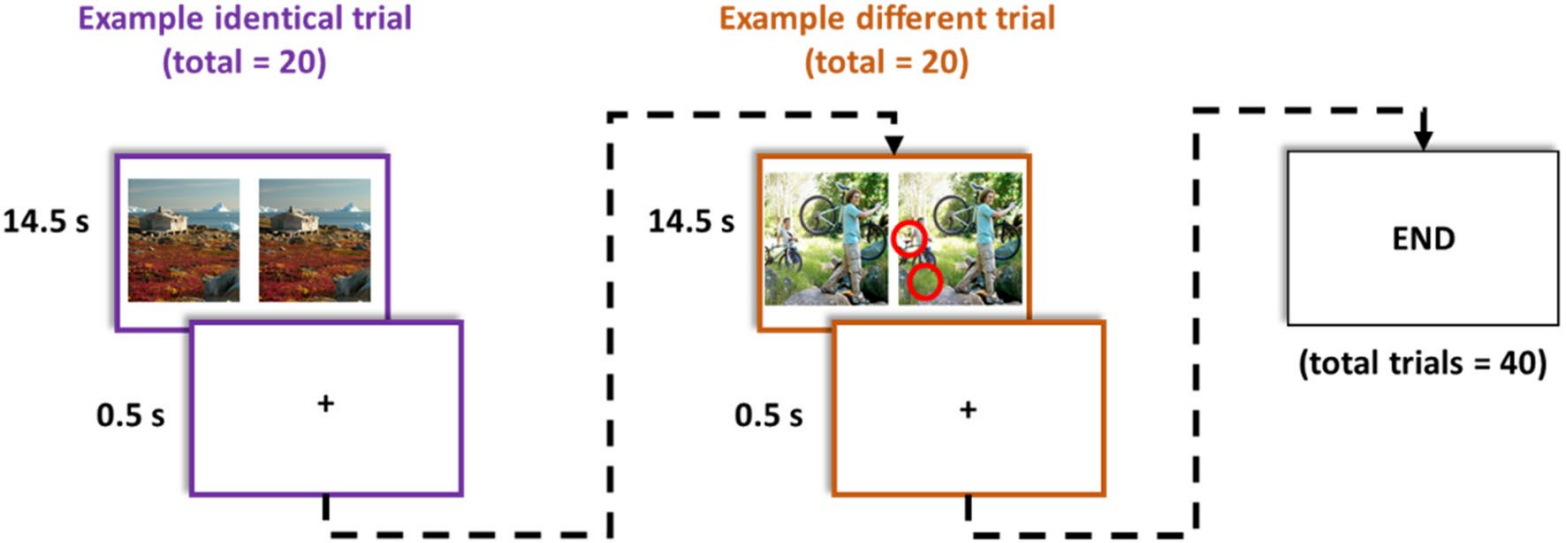
Scene discrimination task. Participants completed a total of 40 scene discrimination trials in this task. Each trial was 14.5 s in duration and separated by inter-stimuli intervals that were 0.5 s long. A different pair of photos of complex real-world scenes (e.g., countryside) was presented in each trial. Presented photo pairs were either identical (‘identical’ trials, total = 20) or contained two subtle differences (‘different’ trials, total = 20). Participants were required to search for possible differences between the two photos and to press the ‘spacebar’ on the computer keyboard when a difference was discovered. Red circles show the location of differences in the example of a different trial. The content of the identical and different scenes was visually and semantically different from the photos of everyday items in the long-term memory test. Photos were a modified version of a ‘spot-the-difference’ game designed initially as a filler task in memory consolidation paradigms (Dewar et al.^35^; Craig and Dewar^16^).

Figure 3 shows an overview of scene discrimination task procedure. Participants completed this task during the 10-min filled delay phase of the long-term memory test and was a modified version of a ‘spot-the-difference’ game designed initially as a filler task in memory paradigms^16,35^. Participants completed a total of 40 trials. Trials were 14.5 s in duration. Before each trial, a cross (+) appeared in the centre of the screen for 0.5 s. Each trial comprised presentation of a pair of photos of complex real-world scenes (e.g., countryside or city scene; see Fig. 3 for examples). The two photos were identical or contained two discrete differences. Participants were instructed to search for differences and press the ‘spacebar’ on the computer keyboard when a difference was discovered. If one difference was found, they were asked to continue searching until they found a second difference, at which point they should again press the ‘spacebar’. If they found both differences, they were asked to continue attending to the photos until new photos appeared. There was no overlap between these photos and those in the long-term memory test.

Two scores were calculated from participants’ responses in the scene discrimination task: (i) a raw discrimination score and (ii) a corrected discrimination score. The raw discrimination score reflects the proportion of correct ‘different’ responses to trials that contained subtle differences between photo pairs (total number of ‘different’ trials = 20). The corrected discrimination score reflects the proportion of correct ‘different’ responses while considering response bias [(proportion of ‘different’ responses to different pairs) minus (proportion of ‘different’ responses to identical pairs)].

#### Questionnaires on sensory imagery and autobiographical memory

At the end of the study, participants completed three established questionnaires: the *Vividness of Visual Imagery Questionnaire (VVIQ)*^17^ was included to explore possible group differences visual imagery^36,37^. In addition, the *Plymouth Sensory Imagery Questionnaire (PSIQ)*^19^ was included to investigate possible differences in imagination aside from imagery, for example, touch and taste. Finally, the *Survey of Autobiographical Memory (SAM)*^18^ explored possible self-reported differences in everyday memory, over and above specific group differences in visual detail memory (as measured through the visual long-term memory test). There was no time limit on questionnaire completion.

For all questionnaires, we investigated whether scores related to performance in our long-term memory test.

### Statistical analyses

Analyses were performed using JASP (Version 0.10.0.0), with the alpha level set to 0.05. ANOVAs with between-subject factor group (hearing vs deaf) were performed to examine possible group differences in background measures, tests, and questionnaires. These ANOVAs were conducted with and without age as a covariate. A Fisher exact test was performed to examine possible group differences in gender ratio. Pearson correlations for individual groups were used to explore trade-offs between response time and test accuracy and relationships between our long-term memory test and other measures.

## Supplementary Information

Supplementary Information.

## Data Availability

The datasets generated during and/or analysed during the current study are available in the Open Science Framework repository, https://osf.io/rhs4p/.
