# Supplementary material for: Evidence for superior encoding of detailed visual memories in deaf signers
Source: Sci Rep. 2022 May 31;12:9097. doi: 10.1038/s41598-022-13000-y (PMC9156778; doi:10.1038/s41598-022-13000-y)
Supplement: Supplementary file 1 — Supplementary Information. [file 41598_2022_13000_MOESM1_ESM.docx]

**Evidence for superior encoding of detailed visual memories in deaf signers**

**Supplementary Information**

**Supplementary methods**

***Short-term memory test***

Participants completed a total of 45 trials in this computerised test. In each trial, a photo of an everyday item was presented for two seconds. Participants were asked to pay attention to the presented item and understood that their memory for the item would be tested shortly after its presentation. After two seconds, the presented item disappeared, and a filler photo of a cityscape scene appeared for two seconds. The same filler photo was used in all trials. Participants’ memory was then probed. They were again presented a photo of an everyday item. However, the photo was either: (i) identical to the recently presented photo (old targets, N = 15), (ii) subtly different to the recently presented photo (similar lures, N = 15), or brand new and very different to the recently presented photo (new foils, N = 15). Figure S1 shows example trials. Participants input responses through the computer keyboard (‘z’ = old, ‘v’ = similar, and ‘m’ = new). There was no time limit for responses. Using the same method as in the long-term memory test, we computed two measures of memory performance: (i) a standard recognition score, and a (ii) Lure Discrimination Index (LDI) score.


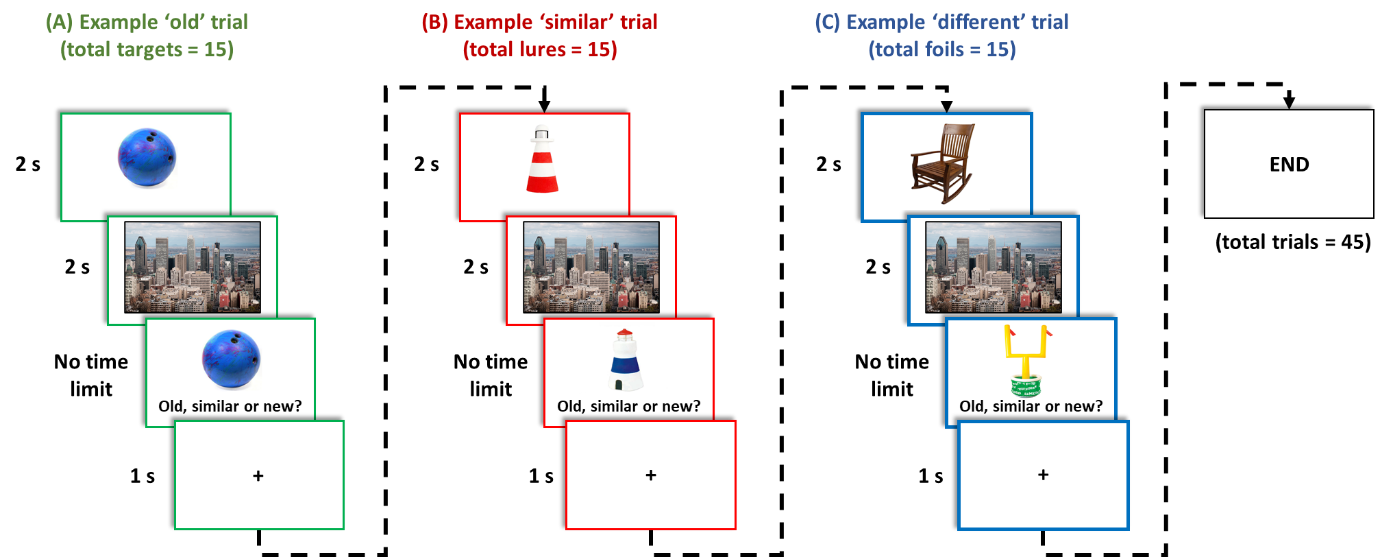


**Figure S1. Short-term memory test procedure.** The test comprised 45 trials. Each trial involved presenting a photo of an everyday item for two seconds, a two-second filled retention interval, and a memory test. In the memory test, participants were presented a photo that corresponded to one of three categories: a photo that was the same as the one presented prior to the retention interval (targets, see A for example), a photo that was similar to the one presented prior to the retention interval (lures, see B for example), or a photo that was different to the one presented prior to the retention interval (foils, see C for example).

***Face discrimination test***

Figure S2 provides an overview of the computerised test procedure, which probed participants’ ability to detect subtle differences in two simultaneously presented faces of the same person. Participants completed 100 trials. Of these trials, 62 contained faces that were identical and 38 contained faces that were subtly different. The face stimuli came from the freely available Glasgow Face Matching Test (GFMT) database (Burton, White, & McNeil, 2010). Participants input responses using the computer keyboard (‘z’ = identical; ‘m’ = different). There was no time limit for participants to respond. Performance in the face discrimination test was measured by computing percentage correct scores for ‘identical’ and ‘different’ trials, as well as an overall percentage correct score.


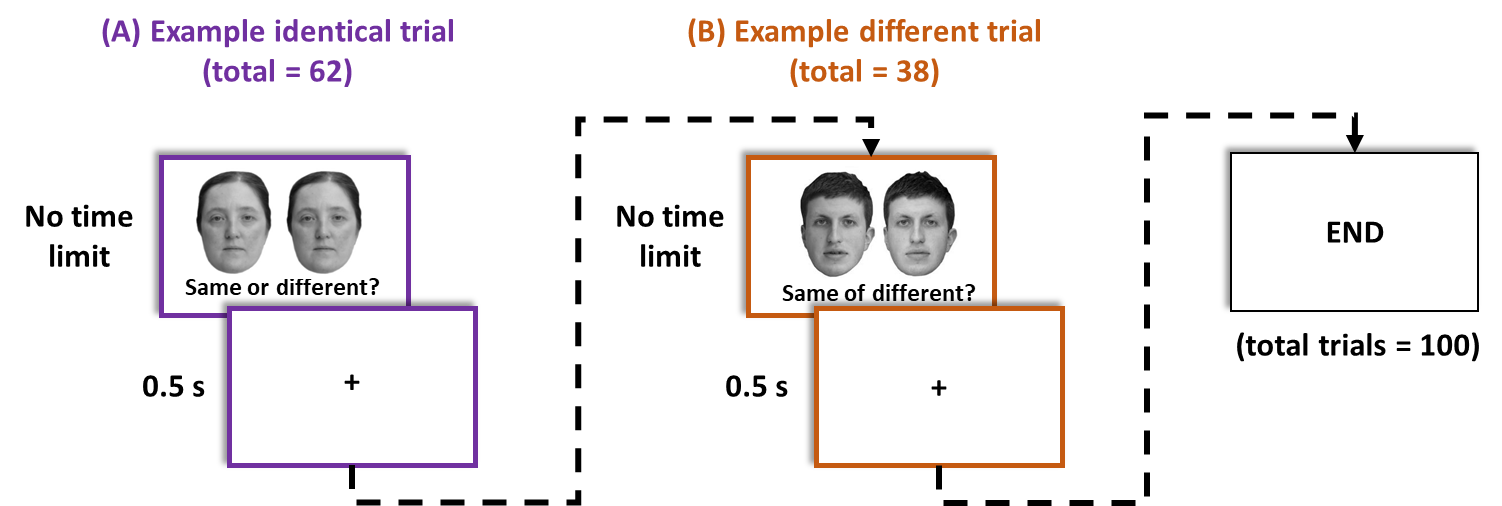


**Figure S2.** Face discrimination test procedure. This test probed participants’ ability to detect subtle differences in two simultaneously presented faces of the same person. Participants completed a total of 100 trials. Of these trials, 62 contained faces that were identical (see A for example) and 38 contained faces that were subtly different (see B for example). The face stimuli came from the freely available Glasgow Face Matching Test (GFMT) database (Burton, White, & McNeil, 2010).

**Supplementary results**

***Short-term memory test***

We found no significant group difference in the standard recognition measure (deaf: mean = 0.94, SD = 0.06; hearing: mean = 0.94, SD = 0.06; F(1,36) = 0.14, p = .722, ηρ² = .004) or LDI measure (deaf: mean = 0.90, SD = 0.10; hearing: mean = 0.90, SD = 0.12; F(1,36) = 0.01, p = .923, ηρ² = .000). Furthermore, performance was near ceiling in both groups.

There was also no overall difference in response times (deaf: mean = 2.21 seconds, SD = 0.80 seconds; hearing: mean = 1.95 seconds, SD = 0.80 seconds; F(1,36) = 1.05, p = .312, ηρ² = .028). Furthermore, we found no significant main effect of item type (targets vs. lures vs. foils) (F(2,72) = 2.62, p = .080, ηρ² = .068). However, we did find a significant interaction between group (deaf vs. hearing) and item type (targets vs. lures vs. foils) (F(2,72) = 6.36, p = .003, ηρ² = .150).

Pairwise comparisons revealed that this interaction was because of a group difference in response times for foils (deaf: mean = 2.64 seconds, SD = 1.20 seconds; hearing: mean = 1.80 seconds, SD = 0.77 seconds; t(36) = 2.55, p = .015), but not targets (deaf: mean time = 1.97 seconds, SD = 1.10 seconds; hearing: mean = 1.82 seconds, SD = 0.66 seconds; t(36) = 0.53, p = .598), or lures (deaf: mean time = 2.03 seconds, SD = 0.78 seconds; hearing: mean = 2.22 seconds, SD = 1.09 seconds; t(36) = -0.63, p = .533).

Pearson correlations revealed no significant relationships between response times and standard recognition or LDI scores (all p > .150).

***Face discrimination test***

There was no group difference in the percentage of correct responses (deaf: mean = 93.00%, SD = 8.00%; hearing: mean = 94.00%, SD = 4.00%; F(1,36) = 0.54, p = .468, ηρ² = .015). This finding held when breaking data down into sets of identical and different trials. There was no group difference in the percentage of correct responses to identical trials (deaf: mean = 91.00%, SD = 12.43%; hearing: mean = 95.00%, SD = 5.00%; F(1,36) = 1.50, p = .229, ηρ² = .040) or different trials (deaf: mean = 95.00%, SD = 6.00%; hearing: mean = 92.00%, SD = 6.00%; F(1,36) = 1.34, p = .254, ηρ² = .036).

We did find a significant main effect of group in response times (F(1,36) = 9.61, p = .004, ηρ² = .210). This was because, overall, participants in the deaf group (mean = 3.30 seconds, SD = 1.65 seconds) were slower to respond than those in the hearing group (mean = 2.02 seconds, SD = 0.70 seconds). A RMANOVA revealed no main effect of trial type (identical vs. different) (F(1,36) = 0.63, p = 0.433, ηρ² = .017), or interaction between trial type (identical vs. different) and group (deaf vs. hearing) (F(1,36) = 1.26, p = .269, ηρ² = .034).

Pearson correlations revealed no significant relationships between response times and the proportion of correct responses to identical trials or different trials (all p > .124).

**References**

Burton, M., White, D., & McNeil, A. (2010). The Glasgow Face Matching Test. *Behavior Research Methods*, *42*(1), 286–291.
